# Author Correction: Improved genome recovery and integrated cell-size analyses of individual uncultured microbial cells and viral particles

**DOI:** 10.1038/s41467-017-02128-5

**Published:** 2017-12-12

**Authors:** Ramunas Stepanauskas, Elizabeth A. Fergusson, Joseph Brown, Nicole J. Poulton, Ben Tupper, Jessica M. Labonté, Eric D. Becraft, Julia M. Brown, Maria G. Pachiadaki, Tadas Povilaitis, Brian P. Thompson, Corianna J. Mascena, Wendy K. Bellows, Arvydas Lubys

**Affiliations:** 1 Bigelow Laboratory for Ocean Sciences, 60 Bigelow Drive, East Boothbay, ME 04544 USA; 2 Thermo Fisher Scientific Baltics, Graiciuno 8, LT-02241 Vilnius, Lithuania; 3 Department of Marine Biology, Texas A&M University at Galveston, Galveston Texas, 77553 USA

*Nature Communications* **8**:84 doi:10.1038/s41467-017-00128-z; Article published online: 20 July 2017

The original version of this Article contained errors in the units of concentration of three reagents listed in the Methods section. In the first sentence of the ‘Cell lysis’ section of the Methods section, the concentration of KOH was incorrectly listed as 0.4 mM and should have read 0.4 M. In the penultimate sentence of the first paragraph of the ‘Optimization and benchmarking of single-cell WGA-X’ section of the Methods, the concentration of dNTP was incorrectly given as 0.4 µM and should have read 0.4 mM. Finally, in the same sentence, the concentration of dithiothreitol was incorrectly given as 10 µM and should have read 10 mM. These errors have all been corrected in both the PDF and HTML versions of the Article.

