# Supplementary material for: Improved genome recovery and integrated cell-size analyses of individual uncultured microbial cells and viral particles
Source: Nat Commun. 2017 Jul 20;8:84. doi: 10.1038/s41467-017-00128-z (PMC5519541; doi:10.1038/s41467-017-00128-z)
Supplement: Supplementary file 1 — Supplementary Information [file 41467_2017_128_MOESM1_ESM.pdf]

File name: Supplementary Information
Description: Supplementary Tables, Supplementary Figures and Supplementary References

File name: Supplementary Data 1
Description: Properties of soil prokaryotic cells: cell diameter equivalent (µm); genomic G+C content (%); phylogenetic classification; classification evidence; gDNA amplification method; number of raw sequence reads; number of contigs >2 kbp; assembly size (bp); and length of the longest contig (bp). Classification evidence designations: CheckM classifications on LoCoS assemblies; Metaxa - CREST classification of 16S rRNA genes that were retrieved by Metaxa from LoCoS assemblies; PCR - CREST classification of 16S rRNA genes that were retrieved by PCR amplification.

File name: Supplementary Data 2
Description: Characterization of viral sequences detected in bacterial SAGs.

File name: Supplementary Data 3
Description: Identities of particles that were sorted from outside the prokaryote gate. Provided are: SCGC SAG codes, putative identities, closest relatives in NCBI nr database (including assembly accession and/or BioProject number), gDNA amplification method (gDNA-A), sequencing type (ST; LoCoS or full-depth), genome assembly size (AS), size of the largest contig (LC), number of contigs, fraction of G+C (%) and the number of paired-end reads.

File name: Peer Review File
Description:

Supplementary Table 1. Genomic characteristics of benchmark strains.

Strain	NCBI GenBank	Phylum	Genome size (kbp)	G+C (%)	Number of chromosomes	Coding nu-cleotides (%)	Assembly status	Source[a]	Reference
Prochlorococcus marinus CCMP1375	NC005042	Cyanobacteria	1,751	36	1	89	finished	NCMA	1
Prochlorococcus marinus CCMP2389	NC005072	Cyanobacteria	1,658	31	1	89	finished	NCMA	2
Synechococcus CCMP 2515	NC008319	Cyanobacteria	2,607	52	1	87	finished	NCMA	3
Escherichia coli K12 DH1	CP001637	Proteobacteria	4,631	51	1	87	finished	ATCC	NA
Meiothermus ruber DSM 1279	NC021081	Deinococcus-Thermus	3,097	63	1	92	finished	ATCC	4
Microbacterium sp	NA	Actinobacteria	NA	NA	NA	NA	NA	GoM	NA
Thalassiosira pseudonana CCMP1335	PRJNA191	Heterokontophyta	32,437	47	24	61	draft	NCMA	5
Ostreococcus lucimarinus CCE9901	PRJNA13044	Chlorophyta	13,205	60	21	75	finished	NCMA	6

NA = data not available.
[a] Strain sources: National Center for marine Algae and Microbiota (NCMA), American Type Culture Collection (ATCC), Gulf of Maine (GoM; new isolate).

Supplementary Table 2. Quality of raw reads of WGA-X and MDA SAGs. Eight randomly selected SAGs of each organism and amplification method were used in this comparison. Provided are means and standard deviations. The p values indicate statistical significance of Student t-test comparisons between WGA-X and MDA SAGs. Only bases with quality score >20 are counted.

Amplification	Organism	Reads mapped[a]	Genome covered[b]	Chimeric reads[c]	Deletions[d]	Insertions[e]	SNPs[f]	MNP[g]	Complex[h]
WGA-X	*E. coli*	100 ± 0%	36 ± 21%	4.4 ± 0.6%	0.22 ± 0.16	0.61 ± 0.45	3.5 ± 2.0	0.01 ± 0.02	0.04 ± 0.06
WGA-X	*M. ruber*	88 ± 33%	39 ± 25%	5.2 ± 1.0%	0.27 ± 0.20	1.74 ± 1.16	2.8 ± 1.5	0.00 ± 0.00	0.03 ± 0.03
WGA-X	*P. marinus*	100 ± 1%	51 ± 27%	4.8 ± 1.3%	0.14 ± 0.14	0.27 ± 0.21	2.0 ± 1.0	0.00 ± 0.00	0.00 ± 0.00
MDA	*E. coli*	100 ± 0%	17 ± 6%	3.5 ± 0.9%	0.07 ± 0.08	0.28 ± 0.10	2.1 ± 0.6	0.00 ± 0.00	0.01 ± 0.03
MDA	*M. ruber*	78 ± 42%	10 ± 9%	7.9 ± 5.0%	0.03 ± 0.04	0.59 ± 0.74	2.1 ± 1.3	0.00 ± 0.00	0.00 ± 0.00
MDA	*P. marinus*	99 ± 0%	66 ± 22%	1.6 ± 0.3%	0.15 ± 0.11	0.40 ± 0.12	2.3 ± 0.7	0.02 ± 0.03	0.01 ± 0.03
**p-value**		0.506	0.135	0.757	0.003	0.059	0.165	0.494	0.197

[a] Fraction of reads mapping to the reference genomes.
[b] Fraction of the reference genome covered at least 1x.
[c] Fraction of chimeric reads.
[d] Frequency of deletions, count per million reads.
[e] Frequency of insertions, count per million reads.
[f] Frequency of single nucleotide polymorphisms, count per million reads.
[g] Frequency of multiple nucleotide polymorphisms, count per million reads.
[h] Frequency of complex artifacts, count per million reads.

Supplementary Figure 1. Quality of *de novo* genome assemblies obtained from SAGs of benchmark microorganisms. Box-plots indicate mean, standard deviation and full range of values. Five million 2x150 bp reads were used in each assembly of prokaryote SAGs (*P. marinus*, *E. coli* and *M. ruber*). Twenty million 2x150 bp reads were used in each assembly of eukaryote SAGs (*T. pseudonana* and *O. lucimarinus*). The following number of replicate SAGs was used in this analysis: eight for randomly selected prokaryote SAGs; five for prokaryote SAGs selected based on the lowest reaction Cp; and three for eukaryote SAGs selected based on the lowest reaction Cp.

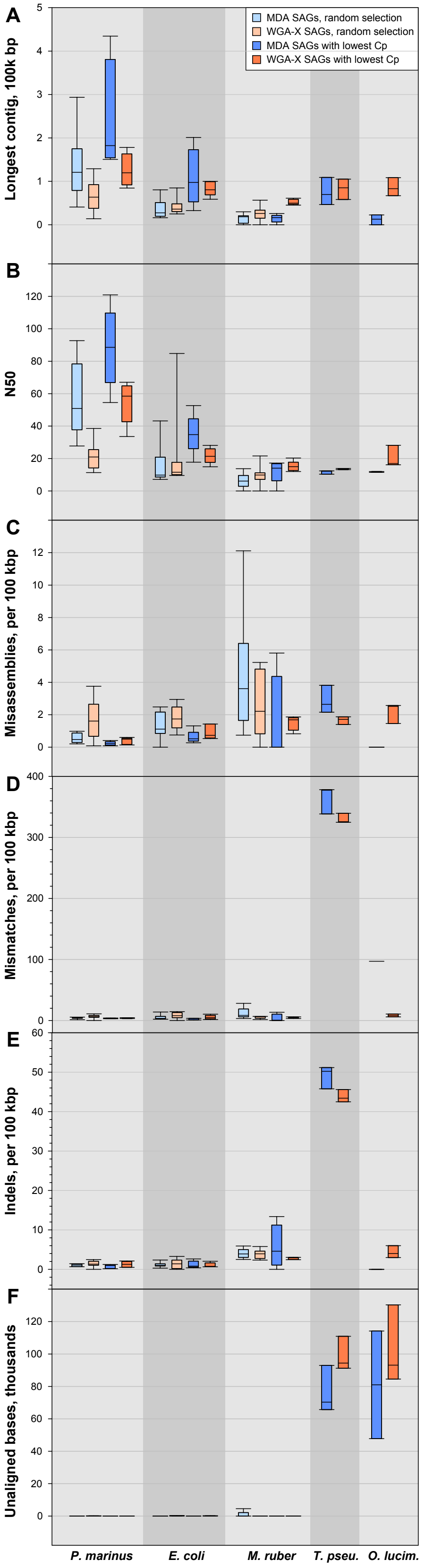

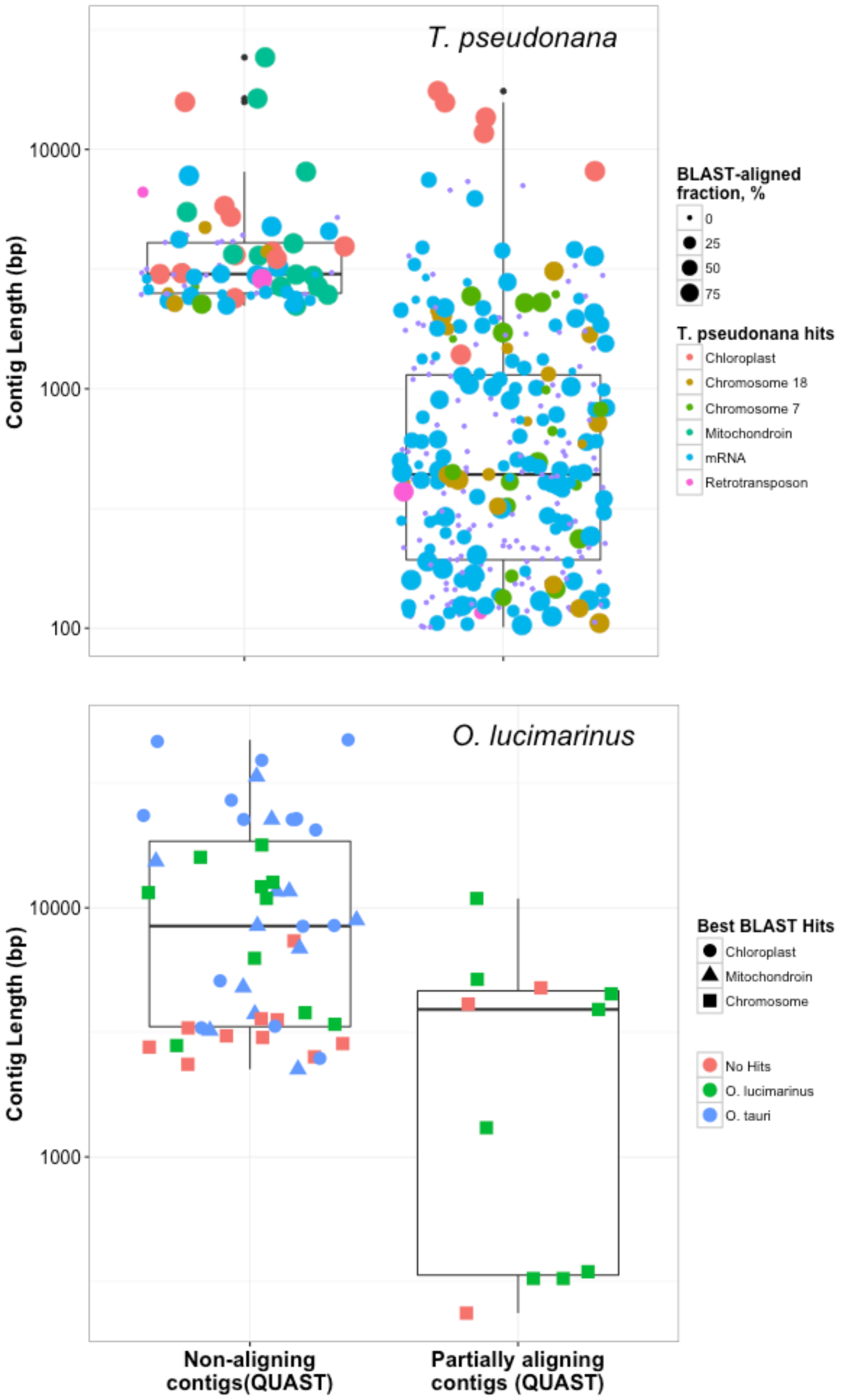

Supplementary Figure 2. Best blast hits to regions of *T. pseudonana* and *O. lucimarinus* SAGs that did not align to the reference genomes with QUAST.

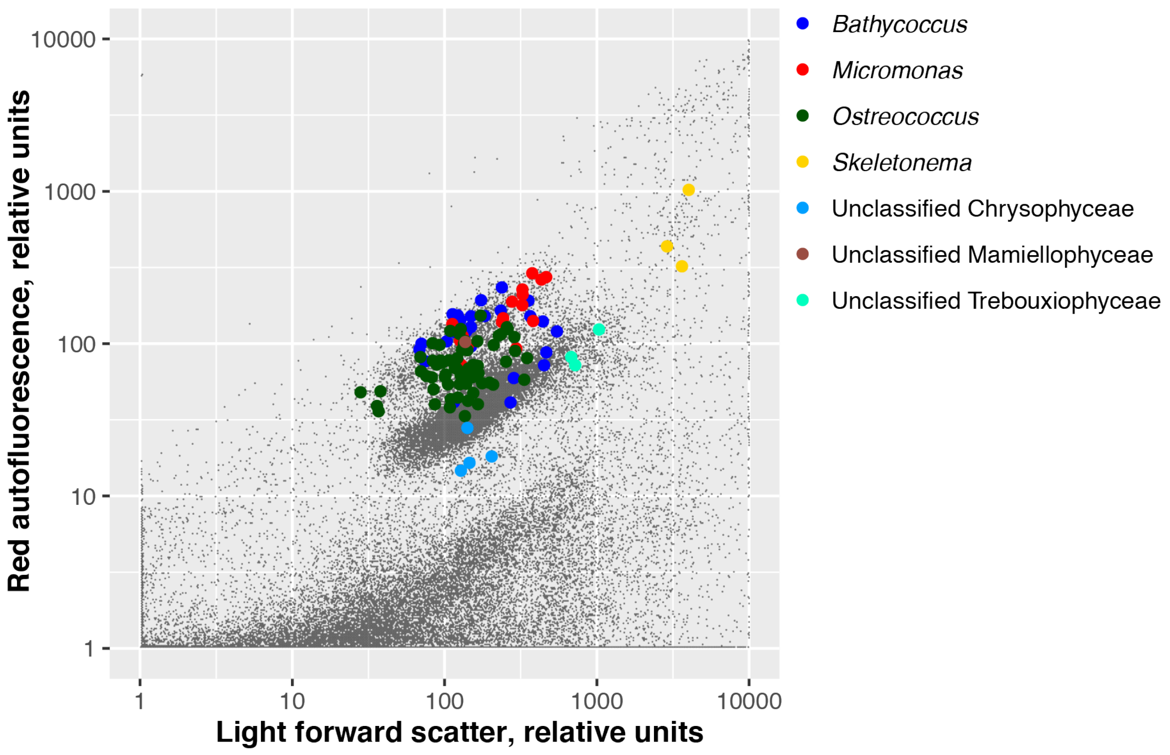

Supplementary Figure 3. Light forward scatter and red autofluorescence of marine microalgae cells.

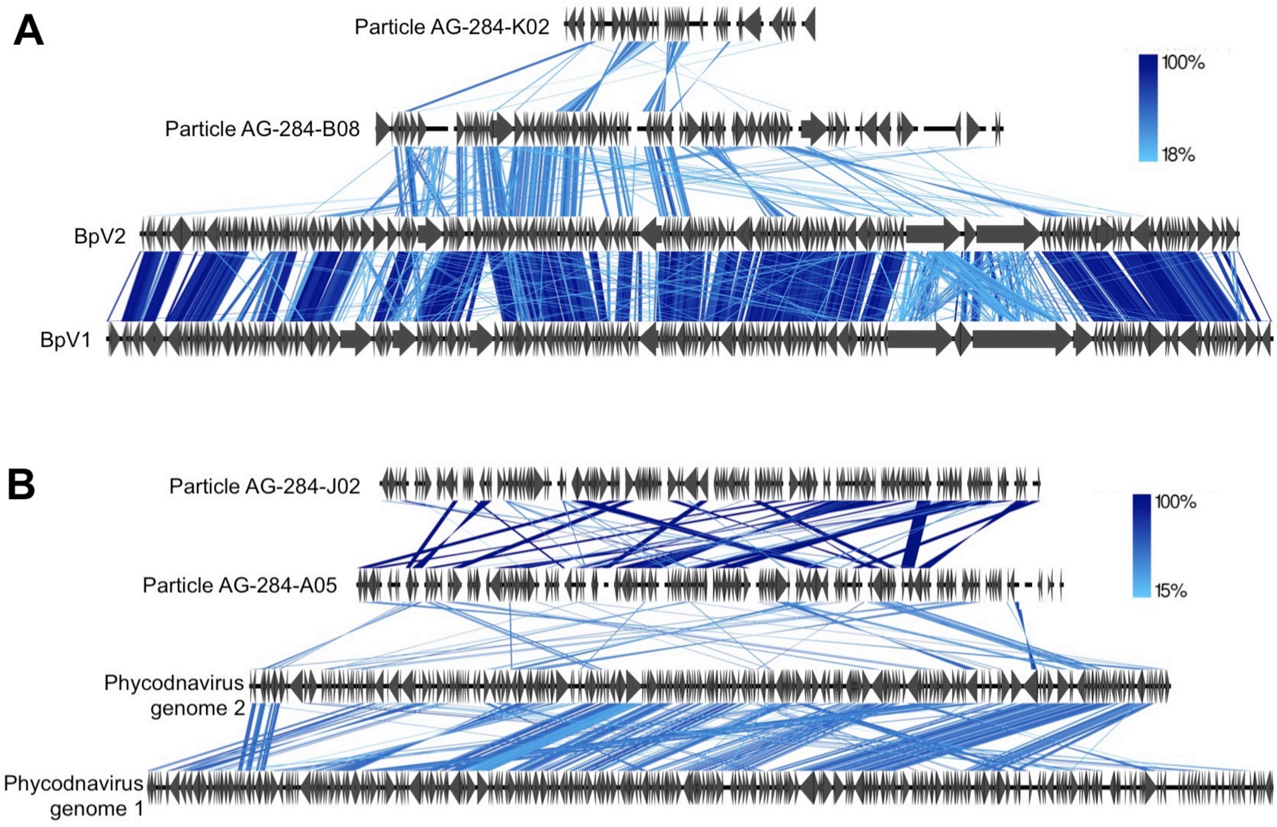

Supplementary Figure 4. Whole genome comparison of individual viral particles from the Gulf of Maine and their closest sequenced relatives. (A) Viral SAGs AG-284-B08 and AG-284-K02 share similarities with *Bathycoccus* viruses BpV1 (NC_014765) and BpV2 (HM004430). Viral SAGs AG-284-A05 and AG-284-J02 are distantly related to large algal viruses and share similarity with *Phycodnaviridae* genomes 1 (Genbank #HQ704802) and 2 (Genbank #HQ704803). Each arrow represents a gene in the direction of transcription. The scale bars indicate peptide sequence identity.

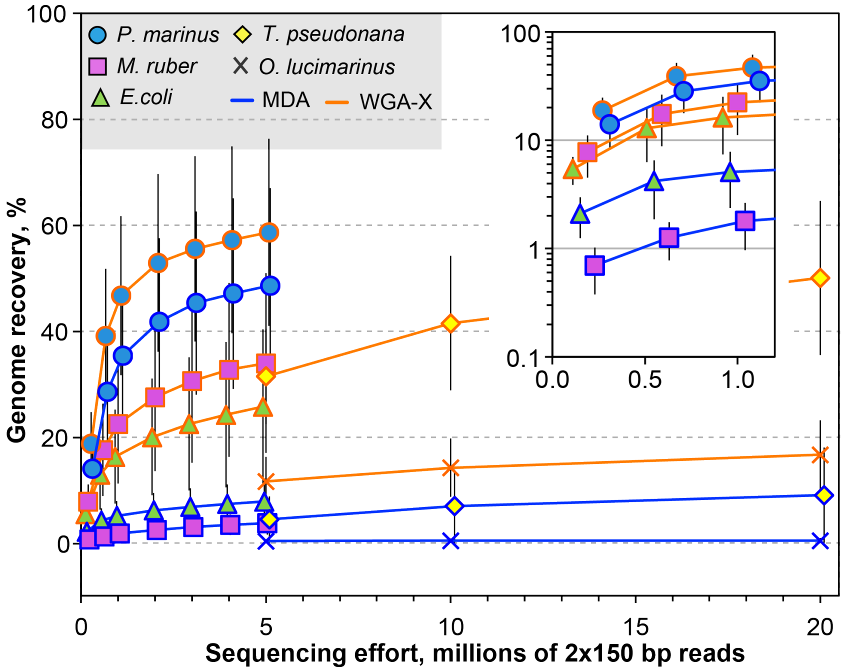

Supplementary Figure 5. Genome recovery from SAGs of benchmark microorganisms as a function of sequencing effort. Paired-end reads of each bacterial SAG were randomly down-sampled to 0.1, 0.5, 1, 2, 3, 4, and 5 millions. Paired-end reads of each eukaryote SAG were randomly down-sampled to 5, 10 and 20 millions. Note that data markers are slightly offset on the horizontal scale, in order to better discriminate standard deviation bars among the treatments. The inset provides a better view of results at low read numbers. Displayed are averages and standard deviations, which were estimated from eight randomly selected SAGs in the case of bacterial SAGs, and from three SAGs with the lowest reaction Cp values in the case of eukaryote SAGs.

SUPPLEMENTARY REFERENCES

1. Dufresne, A. et al. Genome sequence of the cyanobacterium Prochlorococcus marinus SS120, a nearly minimal oxyphototrophic genome. *Proceedings of the National Academy of Sciences of the United States of America* **100**, 10020-10025 (2003).
2. Rocap, G. et al. Genome divergence in two Prochlorococcus ecotypes reflects oceanic niche differentiation. *Nature* **424**, 1042-1047 (2003).
3. Palenik, B. et al. Genome sequence of Synechococcus CC9311: Insights into adaptation to a coastal environment. *Proceedings of the National Academy of Sciences of the United States of America* **103**, 13555-13559 (2006).
4. Tindall, B.J. et al. Complete genome sequence of Meiothermus ruber type strain (21 T). *Standards in Genomic Sciences* **3**, 26-36 (2010).
5. Armbrust, E.V. et al. The genome of the diatom Thalassiosira Pseudonana: Ecology, evolution, and metabolism. *Science* **306**, 79-86 (2004).
6. Palenik, B. et al. The tiny eukaryote Ostreococcus provides genomic insights into the paradox of plankton speciation. *Proceedings of the National Academy of Sciences of the United States of America* **104**, 7705-7710 (2007).
